# A Greener Vision for P–C Bond Formation

**DOI:** 10.1021/acscentsci.3c00943

**Published:** 2023-08-10

**Authors:** Kelsie E. Wentz, Rebekka S. Klausen

**Affiliations:** Department of Chemistry, Johns Hopkins University, 3400 N. Charles Street, Baltimore, Maryland 21218, United States

In this issue of *ACS Central Science*, Cummins and co-workers report the mechanochemical phosphorylation of acetylides, enabling a “greener” phosphorus industry and suggesting new disconnections in organophosphorus synthesis.^1^ Organophosphonate compounds are not only widely used as pharmaceuticals, flame retardants, and more but also play a prominent role as reagents in chemical synthesis (e.g., the Horner–Wadsworth–Emmons reaction).^2^

**Figure 1:**
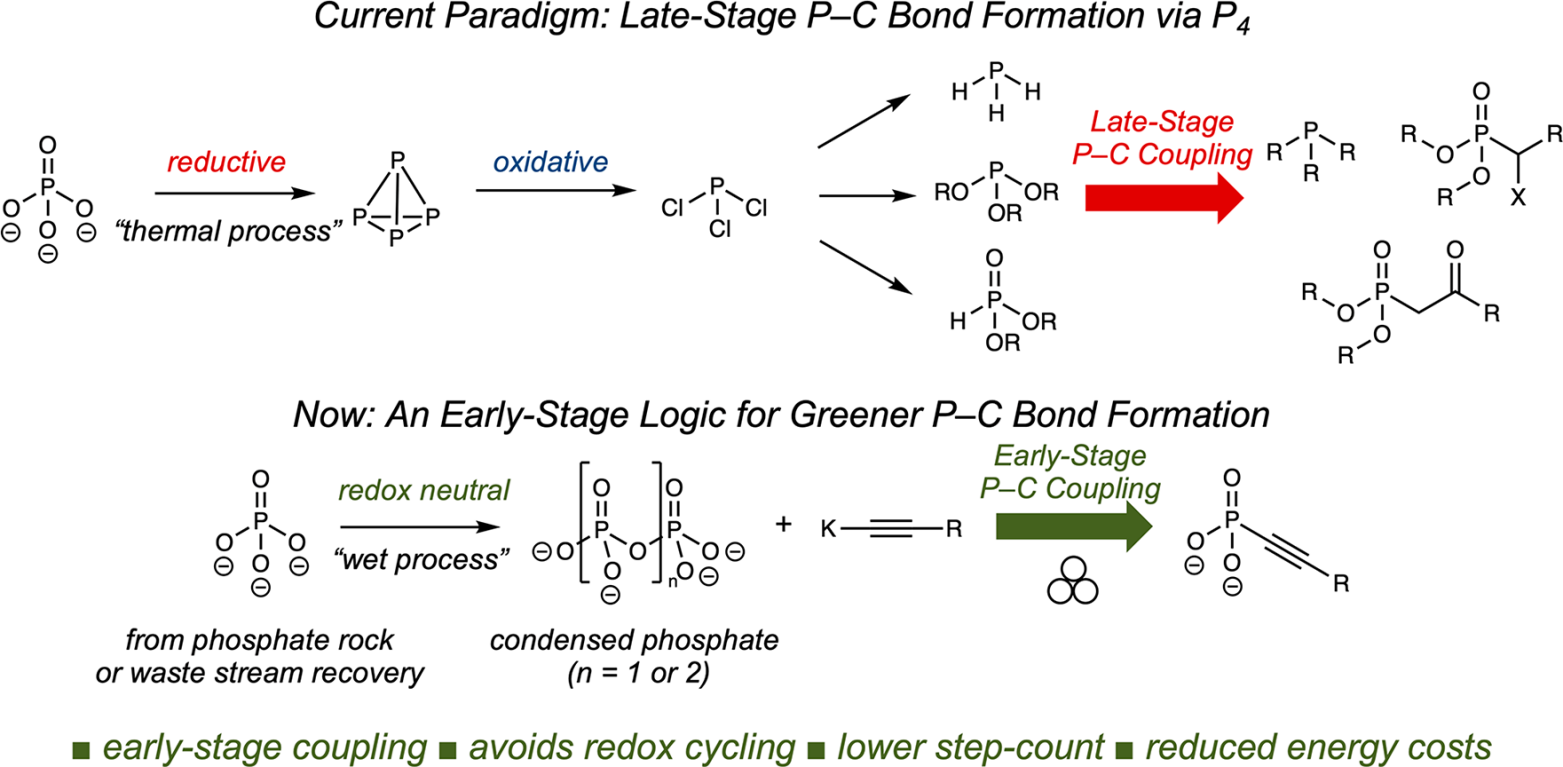
Comparison of P–C bond formation strategies via the current P_4_ paradigm and the mechanochemical strategy reported by Cummins et al.^1^

The current paradigm for organophosphate synthesis necessitates repetitive and energy-costly oxidation state adjustments (Figure 1) as well as late-stage P–C bond formation. Mined phosphate rock is a source of orthophosphate (PO_4_^3–^), which is reduced to white phosphorus (P_4_)^3^ via a high-temperature, high-energy thermal process. P_4_ is then reoxidized to PCl_3_, which is diversified to phosphine (PH_3_), phosphite (P(OR_3_)), or hydrophosphite (HP(O)(OR)_2_), common entry points for P–C bond formation via transformations such as the Michaelis–Arbuzov, Pudovik, and Krabachnik–Fields reactions. In the “wet process”, mined phosphate rock (orthophosphate, PO_4_^3–^) is treated with sulfuric acid to form phosphoric acid (P(O)(OH)_3_) and fertilizers, which upon dehydration yields condensed phosphates such as pyrophosphate (*n* = 1) and triphosphate (*n* = 2). By demonstrating the direct addition of acetylide anions to condensed phosphates, the authors circumvent repetitive oxidation state adjustment and avoid the energy-costs of the thermal process. Critically, they have also invented a new synthetic logic for P–C bond formation.

The authors have previously demonstrated the mechanochemical, solvent-free synthesis of inorganic phosphite (HP(O)O_2_^2–^) via ball-milling of condensed phosphates with potassium hydride (KH).^4^ This direct phosphite synthesis avoided the redox cycling and energy costs associated with P_4_, and the environmental impact of the mechanochemical phosphite synthesis was previously highlighted by Chiu.^5^

Inspired by this breakthrough mechanochemical hydride phosphorylation, the authors sought to expand this redox-neutral process to include carbon nucleophiles. After an initial screen of organometallic reagents, successful P–C bond formation was observed upon ball-milling of triphosphate with potassium phenylacetylide producing phenethynylphosphonate in 33% yield. While the yield is moderate, achieving any significant conversion is remarkable: the multiply anionic condensed phosphonate is a challenging electrophile that would repel negatively charged acetylide. Overcoming this speaks to the powerful impact of mechanochemical activation,^6^ and indeed no solution-phase equivalent of this coupling could be identified in the literature. The moderate yields are also counterbalanced by the significant reduction in step count anticipated by this direct synthesis of organophosphonates relative to traditional methods proceeding via P_4_-derived intermediates. The reduced step count is not only for phosphorus but also for the alkyne, which requires a leaving group for the Arbuzov or related reactions; indeed, a prior synthesis of phenethynylphosphonate via P(OMe)_3_ required four steps from phenylacetylene.^7^

**Figure 2:**
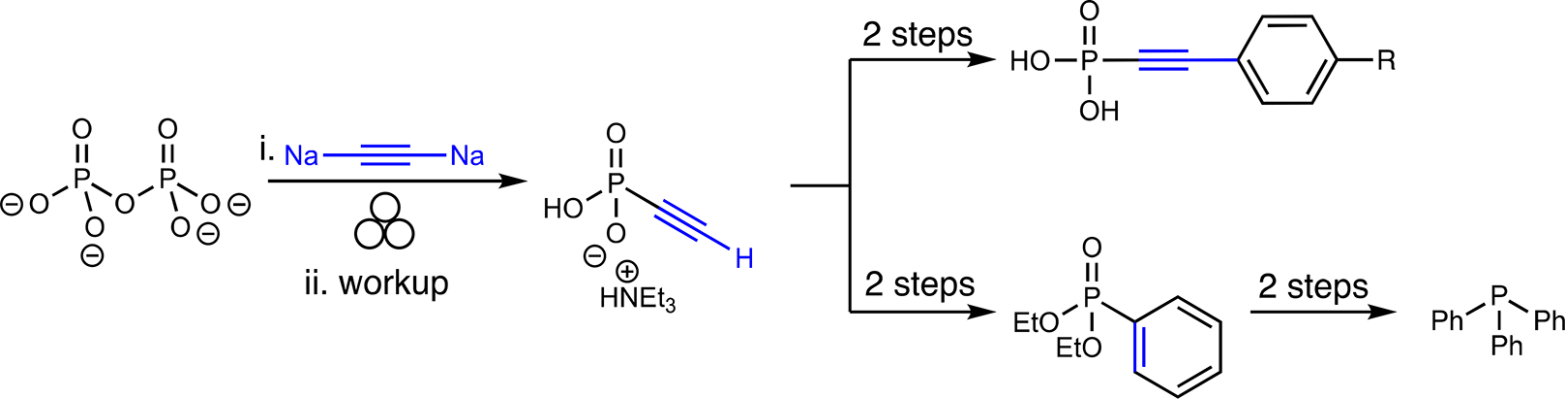
Synthesis of ethynylphosphonate and entry into complex organophosphorus compounds via alkynyl functionalization: synthesis of triphenylphosphine and Sonogashira coupling with functionalized aryl iodides.

The mechanochemical acetylide phosphorylation is applicable to a wide array of aryl and alkyl acetylides, yielding nine other examples [11–32% yields]. With sodium carbide (Na_2_C_2_) as the nucleophile, the authors successfully prepared ethynyl phosphonate in 63% yield (Figure 2). Sonogashira coupling with aryl iodides accessed organophosphonates incompatible with direct acetylide phosphorylation due to limitations in acetylide formation (Figure 2). Additionally, diethyl ethynylphosphonate readily underwent a Diels–Alder ethylene elimination cascade with 1,3-cyclohexadiene, converting the alkyne to a phenyl ring. Subsequent addition of phenylmagnesium bromide and reduction afforded triphenyl phosphine (PPh_3_). Remarkably, this work represents the first synthesis of PPh_3_ without the use of white phosphorus, thereby becoming a foundation for the sustainable production of phosphorus-containing materials.

As impressive as is the scope of the acetylide phosphorylation, a fundamental limitation of using a C2 nucleophile is a lack of direct access to the class of C1 methylphosphonate derivatives. As the authors cite, dimethyl methylphosphonate (DMMP) is a flame retardant. Methylphosphonates are also useful synthetic intermediates. The α-CH bond of an alkylphosphonate is relatively acidic (p*K*_a_ ca. 27),^8^ which makes methylphosphonates an entry point for β-ketophosphonate esters, widely used reagents in organic synthesis, via deprotonation and acylation.^2^ At the same time, the authors have shown several examples of alkyne functionalization—could alkyne hydration obviate the need for methylphosphonate deprotonation?

The widespread usage of phosphorus in fertilizer results in a significant loss of phosphate from soil to waterways,^9^ with negative environmental consequences. Given the potential for the rapid depletion of phosphate rock via this linear process, Cummins et al. considered alternative sources of condensed phosphate. Yeast microorganisms (*Saccharomyces cerevisiae*) converted dipotassium phosphate (K_2_HPO_4_), similar to waste phosphonate that could be recovered from waterways, to biopolyphosphate with an average chain length of 8.1, which under ball-milling conditions was converted to condensed phosphonate suitable for coupling to sodium carbide. These findings indicate that waste phosphonate could be a feedstock for the synthesis of organophosphorus chemicals, enabling a “closed-loop” process.

The mechanochemical acetylide phosphorylation reported by Cummins et al. could significantly reinvent how chemists approach the synthesis of organophosphonates. First, there is a reconsideration of condensed phosphonate as a potential electrophile under mechanochemical conditions. Second, there is the overall reduction in step count enabled by direct P–C bond formation without phosphorus oxidation state adjustment and interconversion of the acetylene to other functional groups. Cummins et al. have demonstrated several exciting functionalization reactions of the alkynylphosphonates, including Sonongashira coupling and Diels–Alder cycloaddition. We look forward to seeing what further alkynyl functionalization might be achievable.

Cummins et al. have rewritten the playbook for P–C bond formation, not only making the chemistry greener and more direct but also changing how chemists might think about the step sequence in organophosphonate synthesis.
